# Dynamical properties of gene regulatory networks involved in long-term potentiation

**DOI:** 10.3389/fnmol.2015.00042

**Published:** 2015-08-07

**Authors:** Gonzalo S. Nido, Margaret M. Ryan, Lubica Benuskova, Joanna M. Williams

**Affiliations:** ^1^Department of Computer Science, University of OtagoDunedin, New Zealand; ^2^Brain Health Research Centre, University of OtagoDunedin, New Zealand; ^3^Department of Anatomy, Otago School of Medical Sciences, University of OtagoDunedin, New Zealand

**Keywords:** long-term potentiation, gene expression, maintenance, synaptic plasticity, memory, dynamic stability, co-expression analysis

## Abstract

The long-lasting enhancement of synaptic effectiveness known as long-term potentiation (LTP) is considered to be the cellular basis of long-term memory. LTP elicits changes at the cellular and molecular level, including temporally specific alterations in gene networks. LTP can be seen as a biological process in which a transient signal sets a new homeostatic state that is “remembered” by cellular regulatory systems. Previously, we have shown that early growth response (Egr) transcription factors are of fundamental importance to gene networks recruited early after LTP induction. From a systems perspective, we hypothesized that these networks will show less stable architecture, while networks recruited later will exhibit increased stability, being more directly related to LTP consolidation. Using random Boolean network (RBN) simulations we found that the network derived at 24 h was markedly more stable than those derived at 20 min or 5 h post-LTP. This temporal effect on the vulnerability of the networks is mirrored by what is known about the vulnerability of LTP and memory itself. Differential gene co-expression analysis further highlighted the importance of the Egr family and found a rapid enrichment in connectivity at 20 min, followed by a systematic decrease, providing a potential explanation for the down-regulation of gene expression at 24 h documented in our preceding studies. We also found that the architecture exhibited by a control and the 24 h LTP co-expression networks fit well to a scale-free distribution, known to be robust against perturbations. By contrast the 20 min and 5 h networks showed more truncated distributions. These results suggest that a new homeostatic state is achieved 24 h post-LTP. Together, these data present an integrated view of the genomic response following LTP induction by which the stability of the networks regulated at different times parallel the properties observed at the synapse.

## 1. Introduction

Living cells are equipped with a robust and yet plastic analog system, which allows them to respond to environmental inputs. Information sensed from the changing environment is integrated and processed by networks of interacting elements to generate an adequate response. Despite the complexity of the underlying mechanisms, some aspects of cellular behavior are of apparent multistable nature, leading to discrete changes that can last for long periods of time. Cell cycle checkpoints, cell differentiation, and apoptosis represent phase transition of multi-component switches that generate robust (and potentially irreversible) transitions (Siegal-Gaskins et al., 2011). From a genetic control perspective, it has been postulated that attractors in the gene and protein expression dynamics, which are the more stable position to which systems tends to evolve, define the cell's character (Kauffman, 1969). Namely, if individual expression values get close enough to the attractor values, these will remain constant even if disturbed. The characterization of the structure of genetic networks from a dynamical perspective using different theoretical methods (Mestl et al., 1997) predicts two broad regimes. The *ordered* regime, robust against perturbations, and a *chaotic* regime, sensitive to perturbations. While robustness is a hallmark of homeostasis, it is reasonable to expect that transitions between cellular states require an enhanced sensitivity. In such scenario, a compromise between robustness and sensitivity could potentially be attained by a rewiring of the network or by the recruitment of different networks.

The change in synaptic efficiency known as long-term potentiation (LTP) represents the cellular correlate for long-term memory in the mammalian brain. From a systems perspective, LTP offers an attractive model of a cellular switch, whereby activation promotes movement to a new cellular state. Just as any other biological switch, LTP accommodates a compromise between robustness to genetic and environmental noise and sensitivity to discriminate meaningful signals. This characteristic is likely to be distributed at different levels of biological organization. For example, LTP requires activation and trafficking of glutamate receptors to the postsynaptic membrane, in addition to protein synthesis and *de novo* gene expression (Abraham and Williams, 2003). Indeed, specific patterns of gene expression have indeed shown to be regulated at different times following LTP induction, and are crucially involved in the maintenance of LTP (Park et al., 2006; Håvik et al., 2007; Ryan et al., 2011, 2012). Specifically, we reported that the networks derived 20 min post-LTP induction, comprised many transcription factors (TFs), including all members of the early growth response (*Egr*) family, and were associated with functions such as development, proliferation, and neurogenesis. By contrast the networks derived at 5 h contained molecules associated with calcium dynamics, dendritogenesis and neurogenesis and in the networks derived at 24 h neurotrophin-NFKB driven pathways of neuronal growth were identified. Our analysis also revealed several mechanisms controlling the temporal shifts in gene expression such as regulation of specific microRNA and histone deacetylases. Thus, the variety of functions held by the genes offers a glimpse of the potential complexity underlying the genomic response to LTP.

The involvement of complex gene networks in the maintenance of LTP suggests that a multistable switch is also present in the structure of the gene regulatory networks recruited at different times following LTP induction. From a systems perspective, we expect that the transition between the pre- and post-LTP homeostatic state is paralleled by the genomic response. We propose that while gene networks rapidly and transiently activated following LTP-inducing stimulation will show a less stable architecture, networks recruited later will exhibit an increased stability, being more directly related to LTP consolidation and post-LTP homeostatic state. Using random Boolean networks (RBN) simulations, we addressed this hypothesis by studying the dynamical stability of networks previously identified (Ryan et al., 2011, 2012). We also use the gene expression profiles provided in these studies to explore the overall co-expression network architecture. Following identification of tightly co-expressed modules, we used functional analysis to investigate the intramodular differential connectivity at different times post-LTP induction. Our results offer an integrated picture of the genomic response following LTP and support the conclusion that a new homeostatic state is achieved 24 h post-LTP.

## 2. Materials and methods

### 2.1. Network topologies

LTP-related gene expression profiles investigated in this study were taken from Ryan et al. (2011, 2012). Briefly, 20 min, 5 h, and 24 h following induction of LTP at perforant path synapses in the dentate gyrus in awake freely moving rats, gene expression profiling was carried out using Affymetrix RAT230.2 microarrays and the functional relationships of the differentially expressed genes (±1.15-fold change; *p* < 0.05; moderated paired *t*-test between the stimulated and control hemispheres) was explored using the network analysis tool Ingenuity pathway analysis, version 7 (IPA) (Ingenuity Systems, USA; https://www.analysis.ingenuity.com). In the present study we analyzed the highest scoring network from each time point (*N* = 35) alongside the yeast transcriptional network (*N* = 30) as a benchmark for RBN modeling (Lee et al., 2002). The yeast transcriptional network represents potential pathways that yeast cells can use to regulate global gene expression. It provides a useful comparison for our analysis for a number of reasons. First, it was constructed by determining experimentally the binding sites of most of the known yeast TFs. In addition to being comprehensive, the yeast network is of similar size to the LTP networks identified by IPA. Finally, it has been used previously in the literature for RBN models (Kauffman et al., 2003; Karlsson and Hörnquist, 2007; Tuğrul and Kabakçıoğlu, 2010). For a node (gene), the number of incoming connections (edges) is called the in-degree of the node and the number of outgoing connections (edges) is its out-degree. The analyses using RBNs were also applied to two different sets of null-hypothesis random networks. First, an ensemble of 100 random networks was generated for each of the 4 biological networks studied (20 min, 5 h, 24 h, and yeast) by preserving the same number of nodes and edges. In order to construct these random networks, pairs of genes are connected randomly with equal probability from the list of *N* = 35 or *N* = 30 genes until the total number of edges of the biological network has been set.

A more stringent control consisted of 4 ensembles of 100 rewired networks constructed such that each of the genes had the same in- and out-degree as the biological network. These networks are constructed by randomly choosing two edges of the biological network and swapping them so that A → B and C → D, become A → D and C → B. Swapping is prevented if either A → D or C → B exist already (Kannan et al., 1997). Hence, these sets of rewired networks preserve not only the number of genes and the total number of interactions, but also the original degree sequence and hence their local connectivity is identical to the biological network. These controls allowed us to discriminate the effects of the network's local structure from the effects of the general topology on the robustness.

### 2.2. Random boolean networks

RBNs represent one of the simplest models for gene regulatory networks (Kauffman, 1969). We assumed the expression of each gene in a network to be only “on” or “off” values (corresponding to transcriptionally active and transcriptionally inactive). If we define the state of the network as the set of values of expression of all the genes, for a network of size *N*, a bit vector *G*(*x*_1_, …, *x*_*N*_) with *x* = 0, 1 suffices to fully describe it. We implemented the connectivity of a given network as a matrix *W* of size *N* × *N* where *w*_*ij*_ = 1 if gene *j* acts upon gene *i*, and *w*_*ij*_ = 0 otherwise.

To model the gene expression dynamics of the network, we allowed the vector *G* to evolve across discrete time steps. We considered the value *x*_*i*_ of the gene *i* at a certain time step *t* to be dependent only on the values at *t* − 1 of the *k*_*i*_ genes that act on it. For each gene in the network, hence, a fixed Boolean function *b*_*i*_ mapped every possible combination of values of the *k*_*i*_ inputs of *i* to an updated value for the gene *i*.

On each interaction, we updated all the values of the state vector *G* synchronously. This means that given a set of values for the state vector *G*(*t*) and a set of Boolean functions *B*, the dynamics of the system are deterministic and the number of possible states for *G* is discrete. Furthermore, it is known that using synchronous RBN dynamics leads to the expression values of the genes to converge into a number of recurring states or attractors. Crucially, these states can be regarded as different homeostatic cell states (Kauffman, 1993). The size and number of these attractors characterize the dynamical stability of a network. Note that while we allowed the values of the genes in the network to evolve over time, we kept the connectivity *W* and the set of Boolean functions fixed.

The choice of the synchronous Boolean approximation was guided by an optimal compromise between both conceptual simplicity and computational feasibility, while still holding the capacity to approximate a general stability characterization of a biological network (Kauffman et al., 2003). Indeed, since the gene networks studied were built based on gene expression profiles that represent averages of gene product, a choice of an asynchronous stochastic schema is difficult to justify.

The networks studied using RBN in the present work (see Section 2.1) do not convey any information on the rules for the interactions that could be used to constrain the sample space of all possible Boolean functions. However, as the present study deals with the comparison of the stability conferred by the architecture of specific networks, it suffices to construct the Boolean functions from a flat distribution. Namely, we opted not to introduce a bias in the outputs of the Boolean functions. This means that for a given Boolean function *b*_*i*_ the probability of the output being one of a particular combination of input values is *P*(*b*_*i*_ = 1) = 0.5.

### 2.3. Dynamical robustness

In general, we consider a stable network one in which small perturbations are not amplified in time but rather converge into the same attractor. In this scenario, arbitrarily changing the values of few genes would not have a dramatic effect—after a few iterations, the perturbed genes would return to their original values. The opposite behavior is also possible. We consider an unstable (or chaotic) network one in which the perturbation of few genes results in a generalized change in expression values after few iterations. To simulate the effect of a perturbation in a network, we first chose a set of initial conditions *G*_*A*_(*t* = 0). We generated an additional instance of the network, with the same initial conditions but shifting some of them [a total of *H*(*t* = 0) genes] to the opposite Boolean value, so that the two instances *G*_*A*_(*t* = 0) and *G*_*B*_(*t* = 0) are, in terms of gene expression, *H*(*t* = 0) far apart. This measure is known as Hamming distance, and is equivalent to the size of the perturbation. We then ran RBN dynamics in parallel for both *G*_*A*_ and *G*_*B*_, with the same Boolean functions for τ time steps. We calculated the new updated Hamming distance *H*(*t* = τ) between *G*_*A*_(*t* = τ) and *G*_*B*_(*t* = τ) to establish whether the perturbation converged into the same values, *H*(τ) < *H*(0), or propagated across the network, *H*(τ) > *H*(0). In the study of dynamical RBN systems, these distinct outcomes are said to fall either on the ordered or chaotic regime, respectively (Fox and Hill, 2001).

Different effects of the perturbations are expected with differing network architectures, Boolean functions or specific initial conditions. As we wanted to characterize the contribution of the topology to the stability of the network, we characterized a network by recording the effects of a perturbation over a large number of initial distances *H*(0), initial conditions *G*_*A*_(*t* = 0) and *G*_*B*_(*t* = 0), and random Boolean functions.

In order to visually represent the average effect of perturbations in a particular network, we plotted *H*(0) (size of the perturbation at the beginning of the parallel runs, x-axis) against *H*(τ) (y-axis), where τ is a small number of discrete steps. Plotting different values of *H*(0) (sampling different perturbation sizes) results in a Derrida plot, a popular tool used in RBNs (Derrida and Weisbuch, 1986). While some network architectures tend to absorb small perturbations and the final Hamming distances *H*(τ) are on average smaller than the initial perturbation, some topologies tended to amplify them—few genes with different values of expression lead to dramatically different network states. Crucially, in the Derrida plots these different behaviors fall in the opposite halves of the plot, with robust architectures represented by curves underneath the diagonal, *H*(0) > *H*(τ), and sensitive architectures above, *H*(0) < *H*(τ) (Fox and Hill, 2001), and a network whose Derrida mapping appears tangent to the diagonal is said to exhibit criticality. Note that choosing small integer values for τ (shorter dynamics) captures the effects of the network's local geometry, while larger values reflect the general characteristics of the structure of the network, since information has more time to spread across the network (Aldana et al., 2003). In practice, the slope for the small *H*(0) region reveals the average outcome of a small perturbation. Curves below the diagonal indicate a tendency toward stability (ordered regime) whereas curves above imply instability (chaotic regime). The diagonal *H*(0) = *H*(τ) represents the transition from order to chaos.

To construct the Derrida plots, for a given network we assigned 1000 random sets of Boolean rules and for each set of randomly generated rules we ran 100 parallel simulations with random initial values, uniformly sampling different values of *H*(0). We did not imply any structure in the Boolean rules and these were randomly generated from a flat distribution without any explicit bias.

### 2.4. Gene co-expression network construction

Using the gene expression profiling data obtained from Ryan et al. (2011) differentially expressed genes were ranked according to the significance of their *p*-value. For computational reasons and to enhance the signal in the data, we used only the top 1700 genes of each of the temporal contrasts. The contribution to the final list of each of the different time groups was equivalent in terms of number of genes, and resulted in a set of differentially expressed genes across early and late LTP of 4804 genes with *p*-values ranging from 7.7 × 10^−6^ to 6.7 × 10^−2^. The total number of genes used for network construction analysis is in the same order of magnitude of other co-expression studies (e.g., Ghazalpour et al., 2006).

For the formation of a gene co-expression network for each of these time points we followed the weighted gene co-expression network analysis (WGCNA) protocols (Zhang and Horvath, 2005) as implemented in the WGCNA package of R software (Langfelder and Horvath, 2008). Briefly, for every pair of genes *i*, *j* the Pearson correlation is calculated and transformed into an adjacency measure with a power function, which serves to further separate the highly co-expressed pairs from the weakly co-expressed pairs. We used the scale-free topology criterion to choose the soft threshold *p* = 5 for the adjacency measure calculation (Zhang and Horvath, 2005). As a measure of connectedness, the topological overlap (TO, Ravasz et al., 2002) was used to compute the similarity between genes, resulting in four undirected weighted networks of the same size (*N* = 4804) but with varying connectivity values of TO—control (unstimulated hemispheres), 20 min, 5 h, and 24 h. TO can be understood as a measure of “agreement” between the nearest-neighbors of two genes. It has been shown to be one of the most biologically meaningful similarity measures used in gene co-expression analysis (Ravasz et al., 2002).

### 2.5. Identification and characterization of gene co-expression modules

For each of the four co-expression networks, we constructed specific gene co-expression modules, clusters of densely interconnected genes. This analysis provided a summary of the networks by reducing their complexity to a small number of modules uncovering potential biological associations. For each of the four co-expression networks, we performed a hierarchical clustering using TO as a similarity measure. The branches of the resulting dendrogram were cut using the default parameters implemented in the WGCNA R package (Langfelder and Horvath, 2008). Modules were considered for further analysis if they contained at least 50 genes. Note that the intersections between the modules are not always empty since module detection was performed independently on each temporal expression dataset. As larger modules in the control and 20 min samples appear to segregate into different sets of genes, we chose to keep all the modules and not to merge overlapping modules (see Supplementary Figure S1). Intramodular functional enrichment was calculated using the topGO R package (Alexa et al., 2006) with with a significance criteria of *p* < 0.01 (Fisher's exact test).

### 2.6. Co-expression network reconfiguration

For each of the 58 modules obtained by WGCNA, we quantified the modular differential connectivity (MDC, Zhang et al., 2013), which corresponds to the ratio of the average connectivity for any pair of module-sharing genes at time *T*_1_ compared to that of the same genes at time *T*_2_ where *w*_*ij*_ is the TO between two genes in a given network. The statistical significance was assessed by a false discovery rate (FDR) based on permutation of the gene labels (Zhang et al., 2013). MDC > 1 indicates enhanced co-regulation between genes, whereas MDC < 1 indicates reduced co-regulation. The MDC was calculated for each of the 58 networks for each of the three temporal transitions (control → 20 min, 20 min → 5 h, 5 h → 24 h).

## 3. Results

### 3.1. Dynamic stability of temporal LTP networks

#### 3.1.1. Gene networks recruited earlier following LTP have a more unstable architecture

To test the hypothesis that the gene networks induced more rapidly following LTP *in vivo* show a less stable architecture when compared to the network induced later, we have drawn on data from our previously published microarray data studies Ryan et al. (2011, 2012). To understand the complexity of the gene networks regulated following the induction of LTP, we used Affymetrix DNA microarrays to identify genes differentially expressed at 20 min, 5 h, and 24 h post-LTP induction *in vivo* Ryan et al. (2011, 2012). Analysis of the gene regulatory networks derived using IPA suggested that these networks made an important contribution to the stabilization of LTP. Furthermore, not only were subsets of genes confirmed to be differentially expressed by quantitative qPCR, but also specific microRNA predicted to act as key regulatory hubs within these networks were shown to be differentially expressed in the hours following LTP induction (Ryan et al., 2013; Joilin et al., 2014). Here, we have used RBN modeling to assess the stability of the architecture of the three highest scoring networks as identified by IPA at each time point. These networks were analyzed alongside the yeast transcriptional network as a benchmark for RBN modeling (Lee et al., 2002; Kauffman et al., 2003; Karlsson and Hörnquist, 2007; Tuğrul and Kabakçıoğlu, 2010).

**Figure 1:**
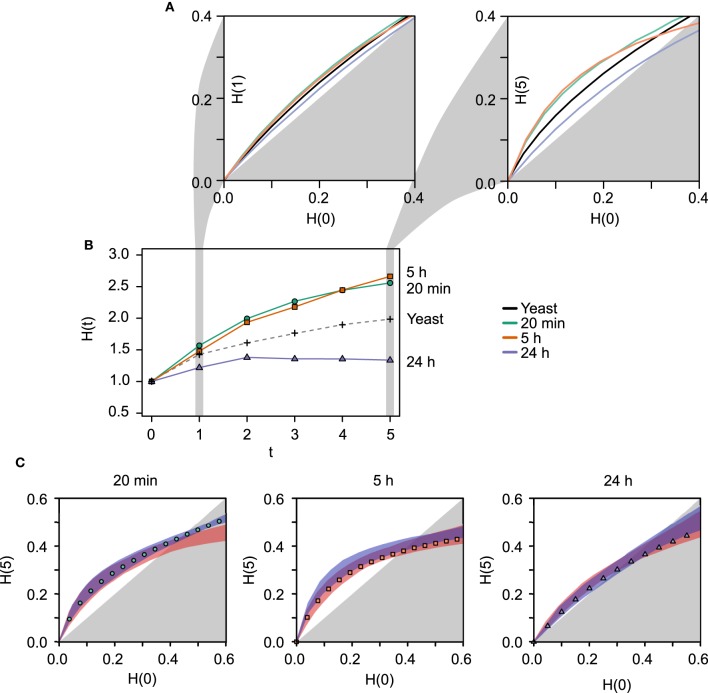
**Results of the RBN dynamical stability analysis**. **(A)** Derrida plots for the LTP networks previously identified by Ryan et al. (2011, 2012) at different times post-LTP induction (20 min, 5 h, 24 h in green, orange, and blue, respectively) and the yeast transcriptional network (black). The left plot corresponds to the initial Hamming distance *H*(0) plotted against the Hamming distance after 1 iteration, *H*(1). Hence, only the nearest-neighbor interactions (local motifs) affect the dynamics. The right plot depicts *H*(0) *vs H*(5), where long-distance indirect influences between genes have an effect on the dynamics. Longer dynamics allow to reveal the influence of the overall network structure on its stability. The earlier networks (20 min and 5 h) are more unstable than the 24 h network, and the curve corresponding to the latter lies near the diagonal of the plot, which represents the border between the chaotic (white background) and the ordered regime (gray background). **(B)** Average time evolution of perturbed fixed points starting from Hamming distance *H*(0) = 1. This small difference tends to be amplified in these biological networks. The latest network recruited following LTP induction (24 h, blue), shows a less pronounced tendency to amplify the perturbation. Furthermore, from *t* = 2 to *t* = 5 the Hamming distance shows a slight decrease. The yeast transcriptional network (black dashed line) lies between the earlier LTP networks and the 24 h. **(C)** Derrida plots for each LTP network and to ensembles of random networks. The same stability profile shown in **(A)** for *H*(0) *vs H*(5) is shown separately for each of the temporal networks. The range of stability exhibited by the two ensembles of random networks (red shade: same number of nodes randomly connected by the same number of edges; blue shade: same number of nodes, same number of edges, and same in- and out-degree). These contrasts allow to isolate the effect of the specific degree sequence from the effect of the average degree (blue shade vs. red shade). In addition, it shows that if an evolutionary constraint were to act on the degree sequence, the real networks choose the less unstable option among all the possible network architectures with the same degree sequence (namely identical local motifs, blue shade). Each point in the plots is the average over 1000 random rule assignments for 100 random initial conditions (increasing these numbers has no effect on the results). Shades for random networks (red) and rewired networks (blue) correspond to the ranges observed using 100 topologies for each. Hamming distances are normalized by the number of nodes.

Consistent with our hypothesis, the output of the RBN analysis (Figure 1) demonstrates that the network identified 24 h following LTP induction is considerably more ordered than either of the earlier networks (20 min and 5 h) or the RBN benchmark, the yeast transcriptional network. The curve corresponding to the late (24 h) network lies underneath the others, which means that the average outcomes of perturbations to the gene expression levels do not spread across the network to the same extent (Figure 1A; τ = 1). This observation is even more apparent if the simulations are evolved for more iterations, allowing the new values for the gene expression to be used as inputs for next iteration (Figure 1A; τ = 5) before plotting the Hamming distances *H*(0) *vs H*(τ). This amplification of the differences between the temporal networks with longer dynamics indicates that both local motifs and long-distance interactions contribute to the differential stability observed between the temporal networks.

The same conclusion can be drawn from the panel depicted in Figure 1B, where the average Hamming distance of changing one random gene [*H*(0) = 1] is plotted at each consecutive time step. In other words, the two network states differing in only one position are independently evolved over 5 time steps and the distances are monitored at each time step. The amplification of the perturbation is clearly less pronounced than the one observed for the other networks, the yeast network lies between the earlier networks (20 min and 5 h) and the more stable 24 h network.

#### 3.1.2. Stability of random and rewired versions of the real networks

Using a similar RBN model, Kauffman et al. (2003) compared the stability of the yeast transcriptional network with networks of the same number of nodes and edges that also preserved the degree sequence (the same sequence of in- and out-going edges); so-called “rewired” networks. The study demonstrated that the yeast network was more stable than these rewired networks, which suggested that an evolutionary pressure may be acting on the network geometry. To assess if that was the case for the temporally specific LTP-related gene networks, we conducted the same analysis by studying the stability of rewired versions of the real networks. We found that the LTP networks lean toward a more ordered regime than their rewired counterparts (Figure 1C), in a manner similar to the yeast transcriptional network analysis. Furthermore, we also analyzed the stability of a less constrained set of random networks that only preserve the number of nodes and the number of edges of the real network (see Section 2). This contrast isolates the effect of the specific degree sequence from the effect of the average degree. The plots indicate that the real networks lie in the less unstable margin of the possible network architectures with the same degree sequence (see Figure 1C). This observation is particularly marked in the cases of the 5 h and 24 h networks, and supports the idea that the stability is not only dependent on the local structural motifs but rather is distributed across the global architecture of the network (Wagner, 2005).

### 3.2. Co-expression analysis

Although analysis of the IPA generated networks has provided validated and biologically meaningful data (Ryan et al., 2011, 2012; Joilin et al., 2014), some limitations are inherent to the methodology. First, potential key interactions may be excluded as the interactions of only 35 genes per network have been considered. Secondly, the architecture of each network is directly dependent upon the information contained within the IPA Knowledge base, a manually curated database, which makes these networks susceptible to false negatives. Thirdly, genes that are modestly but consistently regulated at each time point will be excluded if they do not reach the inclusion criteria at any time. Finally, the analysis is incompatible with the identification of a *control* network, allowing the characterization of a pre-LTP homeostatic state, as networks are based on differentially expressed genes. Thus, to rigorously test our findings that not only are biologically relevant groups of genes regulated following LTP, but that the resultant networks have specific architectural properties and become more stable with time, we used the WGCNA methodology (Zhang and Horvath, 2005) to construct weighted gene co-expression networks based on each of the sample classes (unstimulated hemispheres, 20 min, 5 h, and 24 h stimulated hemispheres). We next assessed the connectivity distributions within these networks.

#### 3.2.1. Identification of co-expression modules

**Figure 2:**
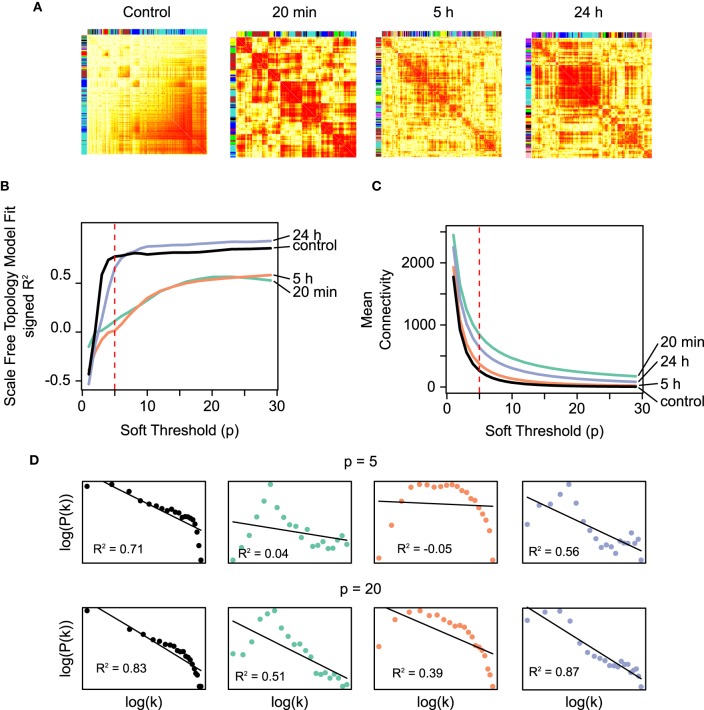
**(A)** The co-expression networks (*N* = 4804) corresponding to the different temporal microarray datasets (control, 20 min, 5 h, 24 h) represented as heatmaps. The darker shade of red represents higher TO values between a pair of genes at that particular time. The TO measure represents the degree of “connectedness” between two genes, and it is based on the adjacency measure calculated from the Pearson correlation (see Section 2). **(B)** Scale-free fit index as a function of the soft-thresholding power *p* for the co-expression networks constructed using the time-course microarray data. The *R*^2^ fit to a scale-free distribution of the unstimulated control and the 24 h networks (black and blue curves, respectively) are both higher and saturate at lower values of *p* than the earlier networks (20 min and 5 h co-expression networks, green and orange, respectively). The latter reaches a saturation only of around *R*^2^ = 0.5. Scale-free networks have been shown to be more robust against small random perturbations, while at the same time are sensitive to specific directed perturbations, which confers them a high degree of sensitivity to meaningful signals. These results are in agreement with the notion drawn from the results using RBNs on the IPA networks. **(C)** Mean connectivity as a function of the soft-thresholding power *p*. While the temporal co-expression networks fall into two different categories according to their scale-free distribution fit, the average connectivity does not show a clear temporal-specific pattern. The dashed lines in the plots indicate the value of *p* = 5, chosen to conduct the module identification. **(D)** Fraction of nodes with degree *k* in the above co-expression networks using *p* = 5 and *p* = 20. These degree distributions are log-transformed both in the x- and y-axes. The black lines represent the linear model fit with the values of *R*^2^.

Co-expression matrices were formed using TO as a similarity measure, which represents the degree of “connectedness” between two genes (Figure 2). The top hubs in the co-expression networks according to their degree (TO with the other genes in the network are shown in Table 1). Within the TO matrices we identified a total of 58 densely connected modules through hierarchical clustering (Control: 9 modules containing between 69 and 2327 genes; 20 min: 9 modules with 64–1184 genes; 5 h: 24 modules with 31–535 genes; 24 h: 16 modules with 42–1164 genes) (see Table 1 and Supplementary Figure S2). To explore the functional relationships of the genes within these modules, modules were tested for Gene Ontology (GO) term enrichment using the topGO R package. We present a brief summary here and a more comprehensive list in Supplementary Material.

**Table 1:** **Top hubs in the co-expression networks according to their degree (TO with the other genes in the network)**.

**Probe ID**	**Gene symbol**	**Gene title**
**20 MIN NETWORK**		
1395900_at	Chtf8	CTF8, chromosome transmission fidelity factor 8 homolog (*S. cerevisiae*)
1385824_at	Cep350	centrosomal protein 350
1381003_at	Ikzf2	IKAROS family zinc finger 2
1386234_at	NA	NA
1391555_at	Ncoa3	nuclear receptor coactivator 3
1388079_at	Cacng8	Ca^2+^ channel, voltage-dependent, gamma subunit 8
1388684_at	Fnbp4	formin binding protein 4
1382979_at	NA	NA
1387435_at	St8sia3	ST8 alpha-N-acetyl-neuraminide alpha-2,8-sialyltransferase 3
1387795_at	Pola2	polymerase (DNA directed), alpha 2
**5 H NETWORK**		
1384230_at	Krtcap3	keratinocyte associated protein 3
1374827_at	Ndst2	N-deacetylase/N-sulfotransferase (heparan glucosaminyl) 2
1383540_at	NA	NA
1384860_at	Zfp84	zinc finger protein 84
1394492_at	RGD1563482	similar to hypothetical protein FLJ38663
1368005_at	Itpr3	inositol 1,4,5-triphosphate receptor, type 3
1371697_at	Pnpla2	patatin-like phospholipase domain containing 2
1368229_at	Sip1	survival of motor neuron protein interacting protein 1
1385928_at	Smad6	SMAD family member 6
1368321_at	Egr1	early growth response 1
**24 H NETWORK**		
1369067_at	Nr4a3	nuclear receptor subfamily 4, group A, member 3
1369398_at	Naaladl1	N-acetylated alpha-linked acidic dipeptidase-like 1
1369255_at	Il1r1	interleukin 1 receptor, type I
1384999_at	Lce1d	late cornified envelope 1D
1371003_at	Map1b	microtubule-associated protein 1B
1369237_at	Slc6a7	solute carrier family 6 (neurotransmitter transporter, L-proline), member 7
1380864_at	NA	NA
1397942_at	Cdc37l1	cell division cycle 37 homolog (*S. cerevisiae*)-like 1
1370641_s_at	Cacna1i	Ca^2+^ channel, voltage-dependent, T type, alpha 1I subunit
1377276_at	Cdk5r2	cyclin-dependent kinase 5, regulatory subunit 2 (p39)

Consistent with our previous analysis, WGCNA identified a number of transcriptional modulators as hubs in the 20 min co-expression modules. Our results stress the importance of the *Egr* family, previously reported to be expressed following LTP induction (Cole et al., 1989; Richardson et al., 1992). In particular, *Egr1* appears among the top 10 hubs of the overall 5 h co-expression network (see Table 1). In addition, *Wt1*, a member of the same family, appears as a key regulator in one of the co-expression modules (see Supplementary Figure S2, module brown_20). Its role as a repressor of the other *Egr* family members (Haber et al., 1991) suggests that it may play an important role in regulating *Egr* gene expression after LTP. Similarly, the *Homer* family of TFs has been implicated in LTP (Kato et al., 1997) and *Homer2* appears as a hub in a co-expression module activated at 20 min and 24 h (see Supplementary Figure S2, module turquoise_24).

A representative GO term for each module is shown in Table 2. Functions overrepresented in the modules identified at 20 min show “positive regulation of endocytosis,” “neuron part and cytoplasmic microtubule,” “axogenesis,” “calmodulin-dependent kinase activity,” and “transcription from RNApolI promoter” among others. At 5 h, “CNS neuron axonogenesis,” “anion homeostasis and synapse assembly” are salient overrepresented functions. Regulation of gene expression is represented by the GO terms “regulation of DNA methylation” and “chromatin DNA binding,” “histone H3-K27 methylation.” Finally, modules identified at 24 h show “neuron projection membrane,” “histone demethylation,” and “response to calcium” among others.

**Table 2:** **Summary of the modules identified by WCGNA with at least 50 genes**.

**Module**	**Size**	**Functional category**	**Top genes by *k*_*TO*_**
cyan_24	97	endosome transport	Nog, Thbd, Zscan10
green_U	206	cation transmembrane transport	Camk4, St6gal1, Slc31a1
black_20	329	(+) reg. of endocytosis	Arhgap27, Kdm5b, Acrv1
brown_24	459	cofactor transporter activity	Mast2, Mlst8, Fgd2
black_24	269	epitelial polarization	Cpn1, Thoc2, Pqlc3
brown_20	730	neuron part and cytoplasmic microtubule	Ddi2, Atp5i, Rab22a
red_20	455	axogenesis	Slc10a5, Tnfrsf17, Slc4a11
yellow_U	222	BRCA1-A complex	Acap2, Dr1, Alpk3
blue_20	779	leukocyte activation	Pias2, Atp6v1b2, Pdzd3
green_20	461	response to axon injury	Igha, Tp53bp1, Tal1
pink_20	78	calmodulin-dependent kinase activity	Lmo2, Pacsin1, Hmox3
yellow_20	724	transcription from RNApolI promoter	Ndst2, Kcnj12, Ptpn7
turquoise_20	1184	oxidoreductase activity	Brpf1, Tsta3, Kdelc1
blue_24	753	reg. of endocrine process	RT1-Da, Mrpl14, Ccnd1
turquoise_24	1164	neuron projection membrane	Ak3, Cacna1i, Rbm4
black_U	145	activation of prot kinase and membrane	Znf609, Cd24, Dab2
pink_24	238	fatty-acyl-CoA binding	Gtf3c6, Ak3l1, Ap2a2
yellow_24	355	proteasomal protein catabolism	Qtrt1, Sh3glb1, Hira
magenta_U	69	integrin binding	Uba6, Samd14, Atrx
pink_U	72	mitochondrial transport and apoptosis	nod3l, Rnasen, Glce
green_24	281	histone demethylation	Gls, Junb, Fam135a
brown_U	691	synapse and reg. of secretion	Alox5, Kcnj4, Dhrs9
greenyellow_24	143	tau-protein kinase activity	Hspb3, Hist2h2be, Hiat1
magenta_24	212	proteasomal protein catabolism	Hectd1, Nans, Sec1
midnightblue_24	86	clathrin-coated endocytic vesicle	Reg3a, Dimt1l, Ctrc
red_24	278	septin complex	Crcp, Cc2d1a, Pdia6
purple_24	175	cAMP-mediated signaling	Epor, Xpnpep3, Fam120b
blue_U	836	dephosphorylation and DNA binding	Gabra5, Cdkn2c, Kl
blue_5H	521	amino acid biosynthesis	Scn11a, Abi3, Clec10a
lightyellow_5H	88	CNS neuron axonogenesis	C1qtnf3, Fbln1, St8sia3
black_5H	237	anion homeostasis and synapse assembly	Slc2a4, Pdzd4
cyan_5H	142	reg. of DNA methylation	Fmod, Fam135a, Ankrd6
magenta_5H	208	progesterone receptor signaling	Mrpl35, Prkd3, Cul5
green_5H	336	GTP-Rho binding and mitochondrion	Prelid2, Prl2b1, Abca8
lightgreen_5H	95	T cell migration	H3f3b, Cnih2, Trps1
yellow_5H	358	chromatin DNA binding	Arglu1, Mccc1, Tmem206
turquoise_5H	535	DNA catabolism	Crhr1, Kdm6b, F8
purple_5H	165	histone H3-K27 methylation	Fgf21, Adcy4, Klhl22
salmon_5H	151	oxidoreductase activity	Hs3st2, Hdac5, Ccdc115
tan_5H	158	MAPK import into nucleus	Asb1, Tpr, Pex5l
brown_5H	458	K^+^ transport and Ras GTPase binding	Dhh, Cog7, Dgki
greenyellow_5H	162	response to Ca^2+^	Flrt3, Cnga1, Adra1d
red_U	178	reg. of GTPase activity	Nppa, Cyp8b1, Igfbp2

Size, representative GO term and top genes according to their degree are reported for each module.

#### 3.2.2. Scale-free distributions are distinctive in the Pre-LTP and 24 h LTP co-expression networks

It is widely accepted that biological networks tend to have connectivity distributions that approximate scale-free distributions (Jeong et al., 2000), which define networks with few highly connected nodes and many sparsely connected nodes (Barabási and Albert, 1999). This structural property may be selected for in biological networks due to its robustness against random perturbations while retaining a high sensitivity to directed signals (Albert et al., 2000). From a perspective in which LTP is considered as a high-level switch, these characteristics are to be expected of the genetic networks associated with the pre- and post-LTP homeostatic states. On the contrary, it is plausible that transient topological rearrangements taking place during the transition exhibit architectures that depart from a scale-free architecture.

The WGCNA methodology transforms the correlation between the profiles of expression of two genes into an adjacency measure using a power function. The degree distribution of the network is hence dependent on the choice of the parameter *p* for the power function. To examine this dependency and characterize the temporal networks in terms of their resemblance to scale-free networks, we plotted the scale-free fit index of each temporal network (control, 20 min, 5 h, 24 h) as a function of the parameter *p* (Figure 2B). The results demonstrate that the networks reach an asymptotic maximum fit at different values of *p*, with the unstimulated control and the 24 h networks saturating earlier (*p* ≈ 5 − 10) than the 20 min and 5 h networks (*p* = 20). In addition, the saturation value of the scale-free fit coefficient is higher for the control and 24 h than for the 20 min and 5 h networks. In summary, the control and the 24 h networks fit to a greater degree to a scale-free distribution, independently of the average connectivity (Figure 2C). Furthermore, a closer inspection to the scale-free model fit for the values of *p* = 5 and *p* = 20 reveals that the earlier networks following LTP stimulation (20 min, 5 h), have few nodes with a low degree (truncated left side of the distribution) (Figure 2D).

#### 3.2.3. Changes in connectivity parallel gene up-regulation following LTP

Interestingly, the co-expression networks corresponding to the later times (5 h and 24ḣ) appear to be more dissociated than the earlier networks (control and 20 min), splitting up into more modules for the same power threshold (see Table 2).

**Figure 3:**
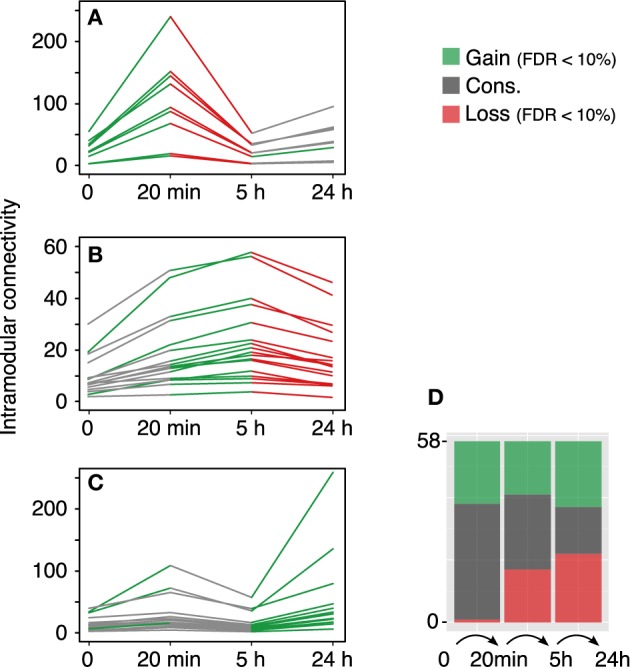
**Module reconfiguration following LTP**. **(A)** Modules identified using the 20 min co-expression data all share a similar trend—they become tightly co-regulated at 20 min to lose the connectivity at 5 h. **(B)** Modules identified at 5 h exhibit a significant decrease in TO at 24 h. **(C)** Modules identified using the 24 h data together with two modules identified with the control data show a significant gain in intramodular connectivity at 24 h. Green lines represent significant gain of intramodular connectivity (MDC > 1, FDR < 10%) and red lines represent significant loss of modular connectivity (MDC > 1, FDR < 10%). Gray lines represent no statistical significance for the MDC. **(D)** Distribution of modules with gain, loss or conservation of intramodular connectivity.

The modular differential connectivity (MDC, Zhang et al., 2013) corresponds to the ratio of the average connectivity for any pair of module-sharing genes at time *T*_1_ compared to that of the same genes at time *T*_2_ where *w*_*ij*_ is the TO between two genes in a given network. We calculated the MDC in the 58 co-expression modules across each temporal transition—control → 20 min, 20 min → 5 h, and 5 h → 24 h. The findings are summarized in Figure 3. While the fraction of modules with a significant increase in MDC is similar along the time samples, the fraction of modules with a significant loss of connectivity increases with time (stacked bars plot, Figure 3D).

The rapid response elicited after LTP induction at the gene expression level corresponds to the transition observed between the control and the 20 min dataset in terms of MDC. Out of the 58 modules, 57 show a gain of connectivity (MDC >1), of which 20 are significant with FDR < 10% (a more strict FDR < 1% gives still a total of 10 significant modules). This marked increase in the average TO connectivity takes place in combination with the documented enrichment in up-regulated genes within the set of differentially expressed genes (Ryan et al., 2011).

Our results, however, highlight a global loss of connectivity between the 20 min and 5 h networks (0.187–0.089 average TO, respectively). Yet, the proportion of modules exhibiting gain/loss/conservation of connectivity is very similar (17/17/24, respectively). More interestingly, the fraction of the modules with a significant loss of MDC corresponds to modules that showed an increase in MDC in the earliest transition. These modules subsequently undertake a loss of connectivity in the final transition to the 24 h phase.

## 4. Discussion

Complex biological networks are expected to maintain a certain level of stability against environmental perturbations. Whereas some classically studied mechanisms such as gene redundancy and epistasis suggest that the dynamical properties of biological networks are restricted to small sets of genes (Sanjuán et al., 2004; Moore, 2005), other authors point in the direction of a “distributed robustness” scenario suggesting that all the regulatory interactions among genes play a role in the dynamical characterization of the network (Shmulevich et al., 2005; Wagner, 2005). These studies suggest that stable, robust attractors for genetic networks may underlie homeostatic cell states. Likewise, the capacity to generate a new phenotype by sensing specific environmental signals represents a crucial property of living organisms. The apparent antagonistic relationship between robustness and sensitivity could potentially be eased by rewiring the network architecture or by bringing other networks into operation. Network architectures recruited transiently during phenotypic transitions do not uphold a homeostatic state and conceivably they are not under a selective pressure to exhibit a stable architecture.

While some biological systems are designed to elicit a graded response to an input, other systems show behaviors that resemble multistable transitions. These systems can range from relatively simple switch-like responses to more complex multistable switches (e.g., Ferrell and Machleder, 1998; Pomerening et al., 2003; Yao et al., 2011). During LTP in particular, the cell state needs to transit from a stable point to another stable point. If this assumption holds for the gene expression profile, LTP induction can be seen as the perturbation needed to shift the gene expression equilibrium to the post-LTP attractor. The neuron has to be able to discriminate the changes eliciting LTP from the environmental noise. From this perspective, LTP represents a cellular mechanism, which operates as a switch. The essentiality of the genomic component for the maintenance of late-LTP suggests that the gene regulatory tier of information processing may have characteristics of a high-level switch. Ultimately, other tiers of regulation acting at different levels of organization (networks of neurons and nerve fiber projections between brain areas) act jointly to create, maintain, and retrieve memories.

### 4.1. Dynamical stability of temporal LTP-related gene networks

Previous work shows that there is a critical temporal window after LTP induction in which a rapid nuclear response takes place (Nguyen et al., 1994). This early phase is in the order of minutes, and is characterized by a rapid up-regulation of gene expression, which persists for many hours. The network identified 20 min after induction represents these early response genes. The set of genes identified 5 h post-LTP induction are not closely related to the 20 min early responding genes, as demonstrated by the expression profiles (Ryan et al., 2012). The nature of this rapid transcriptional response following LTP induction suggests that the underlying mechanisms are facilitating a switch-like response. In this line, Saha et al. (2011) documented recently the presence of stalled RNA polymerase II in LTP immediate early genes, which they interpreted as a mechanism for the rapid neuronal induction observed. However, other mechanisms may be acting jointly at different levels to complement the gene expression trigger.

The 24 h post-LTP induction represents a temporal and functionally different data set, as indicated both by the lack of overlap in gene expression (Ryan et al., 2012) as well as by the fact that mRNA-synthesis inhibitors are only effective in blocking LTP when delivered 4–6 h after stimulation (Vickers et al., 2005). We hypothesized that if the 24 h network was representative of a new homeostatic state brought about by LTP induction, its architecture should display an enhanced stability. While there seems to be an early critical time window of transcription for the induction of late-LTP (Nguyen et al., 1994; Vickers et al., 2005), the functional significance of gene expression at 24 h may be coupled to the activation and coordination of pathways related to growth and/or neurogenesis (Ryan et al., 2012). The predominant downregulation of gene expression observed at 24 h may be partly responsible for reorganizing the transcriptional layout toward homeostasis.

Using RBN modeling, we found that the networks derived in the early time points (20 min, 5 h) by the IPA software were more labile, while the most significant network derived at 24 h was markedly more stable (see Figure 1). Furthermore, the WGCNA showed that the degree of co-expression at the different times evidenced a contrasting distribution of the connectivity. The unstimulated control and the 24 h networks fit to a scale-free distribution fairly well while on the contrary, the co-expression networks corresponding to the 20 min and 5 h datasets displayed a truncated distribution. As higher robustness is expected of scale free distributions (Albert et al., 2000), this observation is consistent with the presence of two different homeostatic states before and after LTP induction, whereas transient topological rearrangements are characteristic of intermediate networks.

Interestingly, this temporal effect on the vulnerability of the networks is mirrored by what is known about the vulnerability of LTP and memory itself. Previous studies have shown that LTP can be reversed within hours of induction, but then becomes resistant to reversal (e.g., Xu et al., 1998; Manahan-Vaughan et al., 2000; Woo and Nguyen, 2002). It is of particular relevance to our studies that this resistance to reversal is dependent on new protein synthesis. Thus, our new data support the conclusion that the LTP-related gene networks contribute to the stabilization of LTP.

These results reinforce the view by which the architecture of the networks is under a selective pressure. Yet the contribution of the structural properties to the overall robustness of biological circuits remains to be further clarified—this tendency toward the stable regime represents only one mechanism yielding robust behavior and does not rule out other genetic mechanisms (Wagner, 2005).

### 4.2. Functional analysis of WGCNA modules

While LTP is considered the gold standard model for the cellular mechanism underlying long-term memories, it is becoming clear that LTP encompasses a family of different processes by which neurons integrate and process the information to change their synaptic weights. For example, it has been argued recently that in the Schaffer collateral-commissural pathway at least three mechanistically different forms of synaptic plasticity co-exist, all N-methyl-D-aspartate (NMDA) receptor-dependent (Park et al., 2014). These forms can overlap partially in time, and the combination of these processes can increase their functional utility. In turn, a specific form of LTP consists of a number of mechanistically distinct phases that operate at different levels of organization and time scales. This should come as no surprise if we recognize that neurons capable of modifying the synaptic efficacy for long periods of time are to overcome the limitations imposed by protein and mRNA half-life, typically in the scale of minutes or hours. In contrast to the TFs, which act in the nucleus, some effector genes exert their functions in the distal axonal and dendritic extensions. The translocation of mRNA granules to the synaptic terminals, for example, carries a significant temporal lag between transcription and translation (Knowles et al., 1996; Steward and Schuman, 2003). The control of gene expression must act in coordination with the different time constraints posed by the different subcellular destinations. It is reasonable to assume that the genes transcribed following LTP are effectively being translated at different times and in different subcellular loci.

While understanding the genomic component underlying LTP has been the focus of recent research using differential expression analysis (Lee et al., 2005; Park et al., 2006; Ryan et al., 2011, 2012), these methods are more error-prone for genes with a large expression variation than co-expression analysis. Potentially, genes which are not detected by differential expression can be detected by co-expression if they activate other genes which change enough to be detected. Arguably, a pair of genes with a high value of co-expression are likely to be forming complexes, pathways, or participate in the same cellular circuits (Eisen et al., 1998). Using WGCNA we found that the rapid increase in gene expression observed by the differential expression analysis is complemented by the increase in intramodular connectivity. Out of the 58 modules identified by WGCNA, only one exhibits a significant decrease in average TO from the control to the 20 min time point, while a total of 20 show a significant increase. This suggests that the rapid genomic response that follows LTP induction does not only involve a marked up-regulation of gene expression, but also a tight coordination of the components that ultimately allow the transition to a new homeostatic cellular state. The transitional 5 h dataset shows a loss of intramodular connectivity that parallels the onset of a general down-regulation of gene expression, similarly to the 24 h co-expression network. We believe that the early phase following stimulation is critical in the onset of the genomic changes that are known to be essential for late-LTP. A fundamental fraction of the genes that are transcribed rapidly after LTP induction may be of crucial importance at later times (Nguyen et al., 1994).

The functional analysis confirms the central role of the *Egr* and *Homer* families in LTP consolidation and maintenance. Changes in transcription are also likely to be driven by the constitutive transcription factor NFKB in a transcription-independent manner. In fact, our results are consistent with its peaks in activity observed in learning paradigms. In agreement with previous studies, we found that the control of gene expression following LTP is, at least to a certain extent, driven by epigenetic changes. Furthermore, it appears that epigenetic control does not work in isolation, but rather in conjunction with other mechanisms (Lubin et al., 2011). Our study identifies *Akt* (protein kinase B) in the 20 min dataset even though its expression does not change significantly. The PI3K-Akt-mTOR is regulated via lipid signaling and its role in LTP may have been overlooked in previous studies (although see Sanna et al., 2002). Finally, while regulators of membrane composition are common across all the datasets, neuronal morphological changes are and the amplification of the ubiquitin-proteasome pathway are characteristic of later stages.

### 4.3. Conclusions

We have presented a view of LTP as a biological process in which a transient signal sets a new homeostatic state that is “remembered” by the cellular systems. Central to this process is the regulation by gene expression, in which the central role played by the *Egr* TFs early after LTP induction was highlighted by differential expression and co-expression analyses. In addition, we found a rapid enrichment in connectivity at 20 min followed by a systematic decrease. This observation provides a potential explanation for the down-regulation of gene expression at 24 h documented by previous studies. From a systems perspective, we have provided evidence that these networks will show less stable architecture, while networks recruited later will exhibit increased stability, consistent with the fact that are more directly related to LTP consolidation. The architecture exhibited by a control and the 24 h LTP co-expression networks fit well to a scale-free distribution, known to be robust against perturbations, whereas the earlier 20 min and 5 h networks showed truncated distributions. Moreover, using the RBN paradigm we have shown that the network derived at 24 h exhibited an enhanced stability when compared to those derived at earlier times post-LTP. This temporal effect on the vulnerability of the networks is mirrored by what is known about the vulnerability of LTP and memory. Taken together, these results suggest that a new homeostatic state is achieved 24 h post-LTP, and defines an integrated view of the genomic response following LTP induction by which the stability of the networks regulated at different times parallel the properties observed at the synapse.

### Conflict of interest statement

The authors declare that the research was conducted in the absence of any commercial or financial relationships that could be construed as a potential conflict of interest.
