# Supplementary material for: Dynamical properties of gene regulatory networks involved in long-term potentiation
Source: Front Mol Neurosci. 2015 Aug 7;8:42. doi: 10.3389/fnmol.2015.00042 (PMC4528166; doi:10.3389/fnmol.2015.00042)
Supplement: Supplementary file 1 [file DataSheet1.PDF]

Supplementary Material: Dynamical properties of gene regulatory networks involved in long-term potentiation

Gonzalo S. Nido [1,2], Margaret M. Ryan [2,3], Lubica Benuskova [1,2] and Joanna M. Williams [2,3*]

[1] *Department of Computer Science, University of Otago, Dunedin, New Zealand*
[2] *Brain Health Reseach Centre, University of Otago, Dunedin, New Zealand*
[3] *Department of Anatomy, Otago School of Medical Sciences, University of Otago, Dunedin, New Zealand*

Correspondence*:
Dr. Joanna Williams
Department of Anatomy, University of Otago, P.O. Box 913, Dunedin 9054, New Zealand, Joanna.williams@otago.ac.nz

The transcriptional regulation of memory

1 SUPPLEMENTARY TEXT

The following sections expand the results obtained using WGCNA on the time-course LTP microarrays.

1.1 REGULATION OF GENE EXPRESSION

WGCNA identified a number of transcriptional modulators as hubs in the 20 min co-expression modules. Our results stress the importance of the Egr family, previously reported to be expressed following LTP induction (**Cole et al.**, 1989; **Richardson et al.**, 1992), in particular, *Egr1* and *Wt1*, a member of the same family. Its role as a repressor of the other *Egr* family members (**Haber et al.**, 1991) suggests that it may play an important role in regulating *Egr* gene expression after LTP.

The *Homer* family is implicated in LTP though its association with the Ras-Erk1/2 pathway. *Homer1* is over-expressed at 20 min and 24 h and has been extensively studied as an LTP immediate early gene (IEG) (see **Brakeman et al.**, 1997; **Kato et al.**, 1997). Likewise, *Homer2* is over-expressed at 20 min whereas *Homer3* is over-expressed at 5 h. It is known that these genes are differentially concentrated in different hippocampal regions (**Shiraishi-Yamaguchi and Furuichi**, 2007). Hence, it is conceivable that they may play important roles at different times after LTP induction. Note, however, that only *Homer2* appears as a hub in a co-expression module. Another important hub is *Shank1*, whose protein recruits Homer to the membrane at the postsynaptic sites (**Sala et al.**, 2001). These findings are in agreement with the role of *Homer1* as an IEG and further suggest that other members of the *Homer* family may be modulating gene expression at other times after induction, possibly even participating in LTP consolidation and maintenance.

The co-expression analysis also highlight the importance of the constitutive transcription factor NFKB, whose activity can be modulated in a transcription-independent manner. Its central role in LTP has been documented in the literature (see **Freudenthal et al.**, 2004; **Meberg et al.**, 1996). Our results suggest that ubiquitin signaling may be of particular importance in the NFKB pathway during LTP. Within the top genes co-expressed at 20 min, the *Zfp91* and the *Siah2* genes encode for E3 ubiquitin-protein ligases indirectly involved in activating the NFKB pathway. In a later co-expression network identified at 5 h, the

genes *Cul5* and *Usp11*, which encode for components of the ubiquitin/protease system are both under-expressed. The latter regulates pathways leading to the activation of NFKB. Other highly co-expressed genes may be regulating the pathway via direct or indirect interaction with NFKB or its inhibitor IKB at different times post-induction, although mainly in the first time point – e.g. *Ubfd1*, *Ncoa3*, *Map3k3*. The *Snip* gene appears as a hub at 24 h, which suggests that the modulation of the NFKB pathway may not be restricted to a short time frame following high-frequency stimulus, consistent with learning-related NFKB activity in the nucleus, which shows a peak immediately after training and a second peak between 6 and 12 h after training (**Freudenthal and Romano**, 2000), both necessary for memory consolidation (**Merlo et al.**, 2002).

1.2 EPIGENETIC CONTROL OF GENE EXPRESSION

The modification of chromatin structure arises as a fundamental mean of gene expression regulation – we find that a substantial number of highly co-expressed enzymes is involved in epigenetic control, DNA methylation/demethylation and post-translational modifications of histones. The inhibition of DNA methyltransferases, has been shown to prevent LTP induction in vitro and to alter the methylation levels in the promoters of Reelin and Bdnf in the hippocampus (**Levenson et al.**, 2006). In vivo, it has been shown that hippocampus-dependent learning can trigger changes in the methylation levels at the *Reelin*, *PP1*, and *Bdnf* gene promoters. These changes were transient and normal levels were observed at 24 h after the training (**Miller and Sweatt**, 2007; **Lubin et al.**, 2008). Furthermore, mice with a conditional knockdown of *Dnmt1* and *Dnmt3a*, two DNA methyltransferases, show impairment of LTP and memory formation *feng2009role*.

Although histone acetylation seems to be less determinant for memory formation, and inhibiting the action of histone acetylases can lead to improve memory formation (**Levenson et al.**, 2006; **Guan et al.**, 2009). An exception may be *Sirt1*. In a work carried out by **Michán et al.** (2010), the authors evidenced the crucial role of Sirt1, a histone deacetylase, in the induction of LTP. Sirt1 was able to inhibit the transcription of miR-134, a microRNA able to bind to CREB mRNA and inhibit its transcription. Furthermore, LTP induction in hippocampal slice cultures from *Sirt1* loss-of-function mice was impaired. Similarly, over-expression of miR-134 resulted in LTP impairment. In the case of the loss-of-function mutation, LTP was restored by the knockdown of miR-134. Furthermore, a recent large-scale study by **Joilin et al.** (2014) documented a generalized and rapid down-regulation of a large number of miRNAs following LTP induction.

In the light of these results, the over-expression observed in the 20 min dataset of the tumor suppressor *Hic1* is difficult to interpret, since it is known to transcriptionally repress *Sirt1* (**Chen et al.**, 2005). The functions of *Hic1*, however, are not restricted to the inhibition of *Sirt1*. In addition, the repression of *Sirt1* requires of the formation of a Hic1/Sirt1 complex, which acts on its promoter region (**Chen et al.**, 2005). Hence, down-regulation of *Sirt1* is directly dependent on its own protein levels, and this pathway may only represent a homeostatic mechanism to prevent over-expression of the deacetylase, which can increase cell proliferation cancer risk (**Liu et al.**, 2009). The *Bcl6* gene, an epigenetic regulator involved in the control of neurogenesis also requires the recruitment of *Sirt1* (**Tiberi et al.**, 2012).

The large number of transcriptional regulators that directly influence chromatin structure and appear as hubs in the co-expression networks identified 20 min post-LTP – e.g. *Phf21a*, *Cxxc1*, *Ring1*, *Setdb1*, and members of the neuron-specific chromatin remodeling complex (nBAF) – suggest that the epigenetic regulation is central to the early stages after the induction of LTP. However, in the 5 and 24 h datasets, other members of the nBAF complex appear as highly co-expressed, together with a significant number of epigenetic regulators, suggesting that the control of gene expression by chromatin modification may be a fundamental process throughout the different stages of LTP.

1.3 REGULATION OF THE KINASE-PHOSPHATASE SYSTEM

The WGCNA provides further evidence for the modulation of intracellular signaling pathways known to be involved in LTP via changes in gene expression. In particular, the modulation of the Ras-Erk1/2 pathway appears to happen mainly in the 20 min dataset. In addition to the discussed *Homer1*, some candidate modulators such as the complex formed by *Ywhab* and *Akirin2* show a high co-expression degree. This complex represses the transcription of *Dusp1*, which is responsible for the inactivation of Erk1/2 (**Owens and Keyse**, 2007). While the *Dusp* family has been implicated in LTP previously (**Ryan et al.**, 2011), our results suggest that *Akirin2* and *Ywhab* may also have an important role despite not showing a significant over-expression in the 20 min dataset.

Perhaps one of the most extensively studied molecular systems involved in LTP is the kinase-phosphatase system. Changes in gene expression also modulate this structure – a large number of kinases and phosphatases were identified across the different co-expression modules. It is well known that protein kinase A (PKA) boosts CaMKII in the CaMK switch in the early stages of LTP by phosphorylation of I1, which in turn inhibits PP1. While the CaMKII-PP1 system may not be necessary for an early-LTP molecular switch as proposed by **Lisman** (1985), the crosstalk between CaM-kinases and phosphatases has a fundamental role in LTP (**Allen et al.**, 2000; **Greengard et al.**, 1999). However, the high co-expression and significant up-regulation at 24 h of a number of genes encoding for phosphate subunits suggests that kinase-phosphatase regulation may also be relevant in the persistence of LTP.

1.4 REGULATION OF THE AKT PATHWAY AND LIPID SIGNALING

The involvement of Akt (or protein kinase B) in LTP in the hippocampus has been suggested to be mediated by phosphatidylinositol 3-kinase (PI3K). PI3K may be required for the expression and maintenance of late-LTP in the CA1 region of the hippocampus (**Sanna et al.**, 2002; **Karpova et al.**, 2006) through extracellular signals in a kinase-independent manner (**Opazo et al.**, 2003). PI3K phosphorylates the D-3 position of the inositol ring of phosphoinositides in the plasma membrane, which converge with phosphoinisitide-dependent kinase-1 (Pdk1) to activate Akt. This convergence is also of a spatial nature, since the activated phosphoinositides are restricted to the membrane. Full activation of Akt is the result of phosphorylation by the mTOR protein kinase. PI3K activity increases in response to calcium/calmodulin, binding to 2-amino-3-(3-hydroxy-5-methyl-isoxazol-4-yl) propanoic acid (AMPA) receptors and driving their insertion during LTP (**Man et al.**, 2003).

Not surprisingly, *Akt1* is central to a module in the 20 min dataset, even though its differential expression is not significant (only moderately over-expressed at 5 h). Interestingly, the analyses performed by **Ryan et al.** (2011) on the same expression data did not identify *Akt* as an important gene in their Network 2, which contains *PI3K*. That same network had as a central hub the platelet-derived growth factor-binding protein homodimer (*PDGF-BB*), known to stimulate the pathway by binding the receptor *Pdgfrb* (**Hanai et al.**, 2006), which we also identified in a 20 min module. Upon ligand binding, Pdgfrb activates the Akt signaling pathway via diacylglycerol (DAG)-inositol triphosphate (IP3) (**Kashishian et al.**, 1992). It can also stimulate the Ras-Erk1/2 pathway (**Yokote et al.**, 1994) and it is involved in the activation of c-Fos transcription (**Kruijer et al.**, 1983).

There is evidence for an important role of IP3 in some types of synaptic plasticity (**Sarkisov and Wang**, 2008). In addition, DAG acts in conjunction with the raise in calcium to activate protein kinase C and facilitate its translocation to the membrane (**Nishizuka**, 1995). The identification in the same module of other members of the DAG-IP3 pathway as *Akt1*, such as *Cds2*, suggests that these pathways may be acting in conjunction. The *Cds2* gene, also over-expressed, encodes for an enzyme that provides CDP-DAG, PI3K precursor of PI (PI→PIP2). Taken together, these findings indicate that lipid signaling actively participates in the induction of LTP, further supporting the notion that *PI3K* gene-expression pathway may be of importance in the early stages of LTP (**Sanna et al.**, 2002).

The regulation of the PI3K-Akt-mTOR pathway by changes in gene expression may also be taking place at 5 h, as evidenced by the identification of the genes *Dab2* and *Plekha1*. These components of the

pathway – *Dab2* is significantly down-regulated at 5 h – are highly co-expressed at 5 h, suggesting that the regulation of this pathway could be coordinating LTP-related molecular events at different time scales.

1.5 OTHER OVERREPRESENTED FUNCTIONS

The 5 h and 24 h networks appear to be enriched in a higher number of genes involved in the ubiquitin-proteasome pathway compared to the earlier networks, suggesting that protein degradation via ubiquitination may be amplified following the early phase of LTP.

Several genes co-expressed at 20 min have functions related with extracellular signaling and anchoring. In particular, the ephrin-receptors identified stress the importance of bidirectional signaling. The involvement of this trans-synaptic signaling in hippocampal synaptic plasticity has been documented in several studies. Using hippocampal slices from gene-targeted mice, the requirement of *Epha4* for the early stages of LTP at the CA3-CA1 synapses was documented. Similarly, *EphB* receptors are thought to be necessary in the postsynaptic membrane for the induction of mossy fiber-CA3 synapses (**Contractor et al.**, 2002). *Epha5*, in turn, is known to be involved in guidance of hippocampal axons during development (**Klein**, 2008).

Gene expression changes also influence the membrane composition. The synthesis of both transmembrane channel subunits and proteins that modulate their trafficking to the membrane is central to synaptic plasticity. Not surprisingly, a large number of genes with membrane functions were identified in the co-expression networks. Indeed, across the three temporal datasets, a substantial number of channels and trafficking modulators appear as hubs. For example, we found that *Gabbr1*, a hub in a 20 min module, was down-regulated – around 15-fold compared to the control – only in the 5 h dataset. This down-regulation may be directly implicated in facilitating LTP induction, as demonstrated by an experimental study carried out in hippocampal slices (**Wigström and Gustafsson**, 1983).

Cornichons, proteins involved in the modulation of AMPA receptor trafficking and gating properties, were also identified together with transmembrane TARPs which have a central role in the control of AMPA receptor gating in the hippocampus (**Kato et al.**, 2010).

An interesting hub identified in the analysis is the gene encoding for Reelin, which has a variety of functions related to microtubule regulation in neurons, neuronal migration, and brain development (**Rice and Curran**, 2001). It also participates in the composition, recruitment and traffic of NMDA receptor subunits in the hippocampus (**Qiu et al.**, 2006), in the generation of dendrites, and in the formation of dendritic spines (**Niu et al.**, 2008). A study carried out by **Weeber et al.** (2002) showed that LTP can be augmented in mice hippocampal slices by perfusing Reelin, whereas this effect is abolished in very low density lipoprotein receptor (VLDLR) and apolipoprotein E receptor (apoER) knockout mice. Both are receptors for apolipoprotein E. The modulatory effect of Reelin on LTP is hence likely to be dependent on both these receptors. This regulation may be related with the recruitment of NMDA receptor subunits. Interestingly there is no significant over-expression of the gene in any of the datasets and this gene was not identified with the classical differential expression analysis with the exception of a 1.75:1 ratio of under-expression in the 5 h dataset.

Neuronal structural changes are known to follow LTP induction. For example, the dendritic spines show morphological changes following the stimulation patterns that elicit LTP. The changes in morphology are associated with the polymerization of actin (**Fischer et al.**, 1998; **Muller et al.**, 2000). The enlargement of spine heads follows closely the pharmacology, amplitude, time course, and spatial localization with synaptic potentiation, and this effect is observed soon after potentiation (**Matsuzaki et al.**, 2004). The formation of new spines and the budding of dendritic filopodia are also modulated by synaptic activity (**Ziv and Smith**, 1996). However, new filopodia or spines require longer times (at least 20 min after the induction of LTP, see **Maletic-Savatic et al.** (1999); **Engert and Bonhoeffer** (1999)).

The actin cytoskeleton is particularly rich in dendritic spines (**Matus**, 2000). The role of actin in LTP seems to be central, as demonstrated by the impairment of LTP that actin inhibitors cause (**Kim and**

Lisman, 1999; **Krucker et al.**, 2000). The inhibition of actin depolymerisation may be the process underlying the changes in the actin cytoskeleton caused by LTP in vivo (**Fukazawa et al.**, 2003). Changes in actin polymerization stand as the main cytoskeletal remodeling mechanism later in the 24 h modules.

Changes in cell morphology play a fundamental role in LTP in vivo. However, morphological changes in the existing synapses are not the only macroscopic changes that occur following LTP induction. The creation of new synapses and neurogenesis are processes linked to LTP and long-term memory (**Snyder et al.**, 2005; **Bruel-Jungerman et al.**, 2006). In fact, the hippocampal region in particular is known to produce new neurons in adults, a process driven by learning processes (see for example **Dayer et al.**, 2003; **Gould et al.**, 1999; **Shors et al.**, 2001; **Raber et al.**, 2004).

Perhaps the most representative gene involved in cell-cycle, encodes for a DNA polymerase subunit. It is over-expressed and central to a 20 min co-expression module. The number of genes involved in regulation of neurogenesis suggests that neuronal proliferation has an important role in hippocampal LTP as suggested by other previous studies (**Ryan et al.**, 2011; **Bruel-Jungerman et al.**, 2006).

Finally, our findings also support the observation that immune response genes are implicated in LTP (**Håvik et al.**, 2007). In particular, we identified immunity-related genes as hubs of some of the modules that exhibit a gain in intramodular connectivity at 24 h.

SUPPLEMENTARY TABLES AND FIGURES

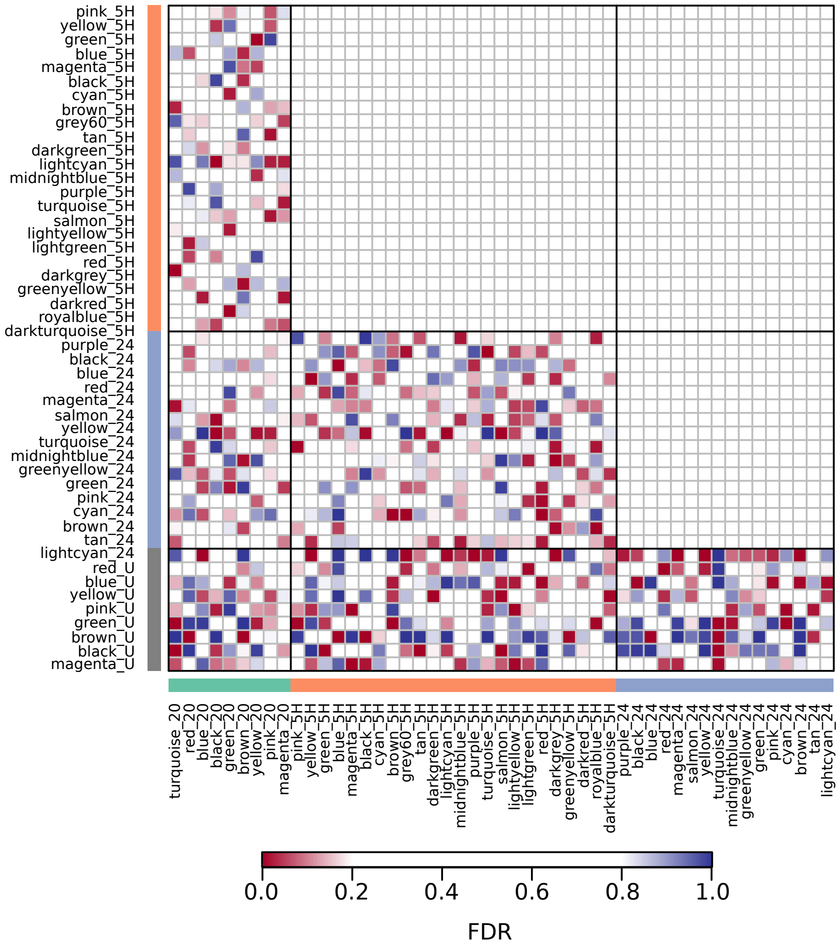

Supplementary Figure 1. Gene overlap between the modules generated from the different expression data corresponding to the different times post-LTP induction. The overlap was normalized by the size of the smallest module. The color in the heatmap represents the FDR of the overlap. Red and blue represent significant overlap and significantly low number of genes in common respectively

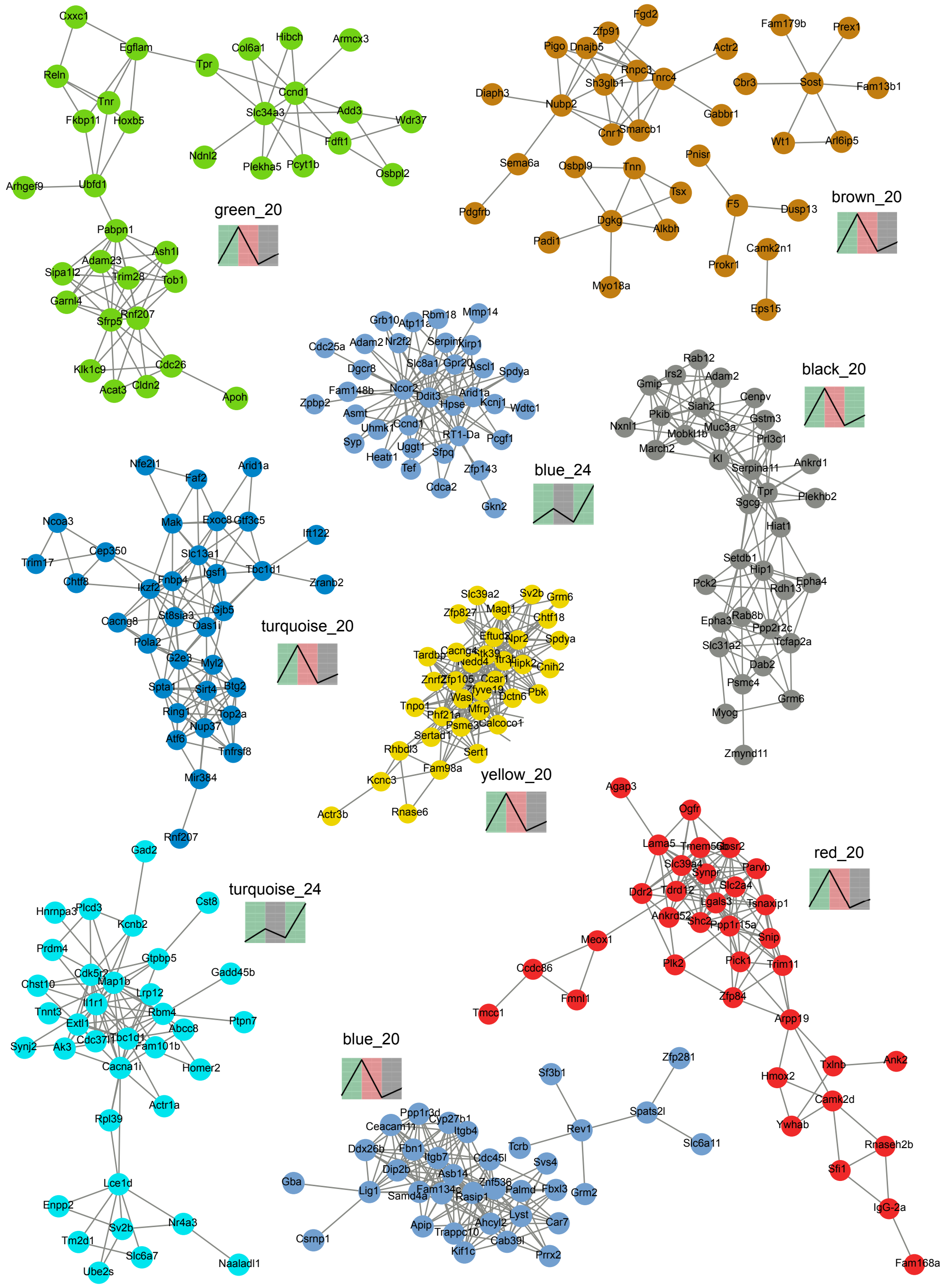

Supplementary Figure 2. Modules identified using WGCNA. Only the top 35 genes for each the modules with more than 500 pairwise interactions with TO $>$ 0.5 are shown. With the exception of blue_24 and turquoise_24, all were identified at 20 min dataset. The panels next to each of the networks show the average TO of the network at the different times (control, 20 min, 5 h, and 24 h post-LTP induction). For each transition, green represents a significant gain of connectivity (MDC $>$ 1; FDR $<$ 10%), red loss of connectivity (MDC $<$ 1; FDR $<$ 10%), and grey conserved connectivity (FDR $>$ 10%)

Table 1: Top Gene Ontoloty terms for each of the modules ($p < 0.01$)

Module	GO Term
black_20	positive regulation of receptor-mediated endocytosis<br>regulation of reactive oxygen species metabolic process<br>regulation of hydrolase activity<br>negative regulation of transcription by competitive promoter binding<br>energy derivation by oxidation of organic compounds<br>ribonucleoside metabolic process
black_24	morphogenesis of a polarized epithelium<br>establishment of planar polarity<br>establishment of tissue polarity<br>post-anal tail morphogenesis<br>acetyltransferase activity<br>canonical Wnt receptor signaling pathway
black_5H	coated pit<br>anion homeostasis<br>neuromuscular junction<br>sarcolemma<br>synapse assembly<br>cholesterol metabolic process
black_U	negative regulation of tissue remodeling<br>membrane budding<br>interleukin-1 secretion<br>regulation of interleukin-1 secretion<br>protein transport<br>response to nitric oxide
blue_20	vitamin metabolic process<br>positive regulation of leukocyte activation<br>positive regulation of cell activation<br>lipid transporter activity<br>water-soluble vitamin metabolic process<br>secretion of lysosomal enzymes
blue_24	regulation of endocrine process<br>endocrine hormone secretion<br>retina development in camera-type eye<br>cellular response to corticosteroid stimulus<br>response to estrogen stimulus<br>steroid catabolic process
blue_5H	cellular amino acid biosynthetic process<br>vacuolar membrane<br>aspartate family amino acid catabolic process<br>aspartate family amino acid metabolic process<br>positive regulation of oxidoreductase activity<br>vacuole
blue_U	nucleus<br>protein phosphatase type 2A complex<br>phosphoric diester hydrolase activity<br>DNA binding<br>sequence-specific DNA binding RNApolII TF activity<br>dephosphorylation
brown_20	cytoplasmic microtubule<br>organic hydroxy compound transport<br>Rho guanyl-nucleotide exchange factor activity

Continued on next page

Table 1 – *Continued from previous page*

Module	GO Term
	monoamine transport<br>microtubule binding<br>cilium
brown_24	cofactor transporter activity<br>heterocyclic compound binding<br>modified amino acid binding<br>organic cyclic compound binding<br>female meiosis<br>cofactor transport
brown_5H	potassium ion transport<br>insulin-like growth factor receptor binding<br>cell body membrane<br>Ras GTPase binding<br>response to nutrient<br>dendrite cytoplasm
brown_U	regulation of secretion<br>plasma membrane<br>regulation of multicellular organismal process<br>cell periphery<br>regulation of system process<br>negative regulation of secretion
cyan_24	gastric acid secretion<br>early endosome to late endosome transport<br>single-stranded DNA binding<br>negative regulation of pathway-restricted SMAD protein phosphorylation<br>acid secretion<br>DNA repair
cyan_5H	regulation of DNA methylation<br>negative regulation of IkB kinase/NFkB cascade<br>DNA alkylation<br>DNA methylation<br>negative regulation of viral transcription<br>osteoblast proliferation
darkgreen_5H	6-phosphofructo-2-kinase activity<br>sugar:hydrogen symporter activity<br>cation:sugar symporter activity<br>phosphofructokinase activity<br>cytoplasmic ubiquitin ligase complex<br>DNA replication factor A complex
darkgrey_5H	M/G1 transition of mitotic cell cycle<br>negative regulation of exit from mitosis<br>regulation of extracellular matrix constituent secretion<br>positive regulation of extracellular matrix constituent secretion<br>glycine metabolic process<br>glycine biosynthetic process
darkred_5H	chondroitin sulfate metabolic process<br>chondroitin sulfate proteoglycan metabolic process<br>lipid phosphorylation<br>phosphatidylinositol phosphorylation<br>aminoglycan metabolic process<br>glycosaminoglycan metabolic process
darkturquoise_5H	negative regulation of protein autophosphorylation<br>response to oxidative stress

Continued on next page

Table 1 – *Continued from previous page*

Module	GO Term
	response to hydrogen peroxide<br>regulation of protein autophosphorylatioon<br>response to reactive oxygen species<br>oxygen transport
green_20	response to axon injury<br>regulation of interphase of mitotic cell cycle<br>response to calcium ion<br>quaternary ammonium group transport<br>reflex<br>alcohol binding
green_24	interaction with host<br>regulation of gliogenesis<br>regulation of organ formation<br>adenohypophysis development<br>embryonic skeletal system development<br>regulation of astrocyte differentiation
green_5H	regulation of amino acid transport<br>steroid binding<br>xenobiotic metabolic process<br>response to xenobiotic stimulus<br>cellular response to xenobiotic stimulus<br>gamma-aminobutyric acid secretion
green_U	outflow tract septum morphogenesis<br>labyrinthine layer morphogenesis<br>embryonic placenta morphogenesis<br>cranial suture morphogenesis<br>craniofacial suture morphogenesis<br>lateral sprouting from an epithelium
greenyellow_24	intrinsic to mitochondrial outer membrane<br>integral to mitochondrial outer membrane<br>superior temporal gyrus development<br>tau-protein kinase activity<br>protein K63-linked ubiquitination<br>protein K48-linked ubiquitination
greenyellow_5H	regulation of myeloid leukocyte differentiation<br>glycoprotein binding<br>macrophage differentiation<br>regulation of macrophage differentiation<br>response to calcium ion<br>protein tyrosine/threonine phosphatase activity
grey60_5H	regulation of branching involved in salivary gland morphogenesis<br>cochlea development
lightcyan_24	regulation of cellular response to stress<br>optic nerve development<br>optic nerve morphogenesis<br>optic nerve structural organization<br>regulation of eosinophil differentiation<br>interleukin-5 receptor binding
lightcyan_5H	pyrimidine nucleotide metabolic process<br>pyrimidine nucleoside triphosphate metabolic process<br>pyrimidine ribonucleoside triphosphate metabolic process<br>pyrimidine ribonucleotide metabolic process<br>pyrimidine nucleoside metabolic process<br>pyrimidine ribonucleoside metabolic process

Continued on next page

Table 1 – *Continued from previous page*

Module	GO Term
lightgreen_5H	regulation of T cell migration<br>positive regulation of T cell migration<br>tumor necrosis factor superfamily cytokine production<br>T cell migration<br>regulation of lymphocyte migration<br>positive regulation of lymphocyte migration
lightyellow_5H	central nervous system neuron axonogenesis<br>establishment of nucleus localization<br>nucleus localization<br>dendritic spine morphogenesis<br>dendritic spine organization<br>dendritic spine development
magenta_20	pattern binding<br>polysaccharide binding<br>protein activation cascade
magenta_24	proteasomal protein catabolic process<br>cullin-RING ubiquitin ligase complex<br>response to virus<br>ubiquitin ligase complex<br>defense response to virus<br>proteasomal ubiquitin-dependent protein catabolic process
magenta_5H	progesterone receptor signaling pathway<br>response to gonadotropin stimulus<br>microvillus<br>blastocyst development<br>epithelial cell differentiation involved in kdney development<br>mesenchymal to epithelial transition
magenta_U	integrin binding<br>inflammatory response
midnightblue_24	clathrin-coated endocytic vesicle<br>chaperone-mediated protein folding<br>mitogen-activated protein kinase binding<br>negative regulation of B cell activation<br>regulation of lymphocyte differentiation
midnightblue_5H	negative regulation of cytokine biosynthetic process<br>neutral amino acid transmembrane transporter activity<br>actinin binding<br>alpha-actinin binding<br>neutral amino acid transport<br>mesodermal cell differentiation
pink_20	calmodulin-dependent protein kinase activity<br>protein serine/threonine kinase activity<br>neural crest cell migration<br>positive regulation of proteasomal ubiquitin-dependent protein catabolic process<br>positive regulation of proteasomal protein catabolic process<br>Golgi vesicle transport
pink_24	fatty-acyl-CoA binding<br>transporter activity<br>sensory perception of chemical stimulus<br>mannose metabolic process<br>cholesterol storage<br>plasma lipoprotein particle assembly

Continued on next page

Table 1 – *Continued from previous page*

Module	GO Term
pink_5H	RNApolII core promoter proximal region sequence-specific DNA binding TF activity involved in negative regulation of transcription<br>RNApolII transcription regulatory region sequence-specific DNA binding TF activity involved in negative regulation of transcription<br>sexual reproduction<br>taurine binding<br>immunoglobulin production<br>activation-induced cell death of T cells
pink_U	mitochondrial transport<br>regulation of intrinsic apoptotic signaling pathway<br>regulation of mitochondrial membrane permeability<br>regulation of release of cytochrome c from mitochondria<br>positive regulation of intrinsic apoptotic signaling pathway.<br>intrinsic apoptotic signaling pathway
purple_24	negative regulation of vascular permeability<br>glycogen granule<br>midbody<br>chemical homeostasis<br>regulation of glycogen catabolic process<br>cAMP-mediated signaling
purple_5H	histone H3-K27 methylation<br>regulation of neutrophil chemotaxis<br>endothelial cell apoptotic process<br>regulation of endothelial cell apoptotic process<br>positive regulation of lymphocyte differentiation<br>regulation of primary metabolic process
red_20	perinuclear region of cytoplasm<br>regulation of axonogenesis<br>dendrite development<br>regulation of neuron projection development<br>regulation of cell projection organization<br>cellular component morphogenesis
red_24	septin complex<br>septin cytoskeleton<br>myeloid leukocyte activation<br>interleukin-23 production<br>regulation of interleukin-23 production<br>negative regulation of interleukin-17 production
red_5H	maternal placenta development<br>regulation of JNK cascade<br>multicellular organismal movement<br>musculoskeletal movement<br>positive regulation of natural killer cell mediated immune response to tumor cell<br>positive regulation of natural killer cell mediated cytotoxicity directed against tumor cell target
red_U	organonitrogen compound metabolic process<br>regulation of GTP catabolic process<br>regulation of GTPase activity<br>positive regulation of GTPase activity<br>cellular amide metabolic process<br>regulation of nucleoside metabolic process

Continued on next page

Table 1 – *Continued from previous page*

Module	GO Term
royalblue_5H	chromatin remodeling<br>carboxypeptidase activity<br>cytosolic part<br>exopeptidase activity
salmon_24	striated muscle adaptation<br>positive regulation of steroid biosynthetic process<br>cGMP biosynthetic process<br>rRNA processing<br>rRNA metabolic process<br>positive regulation of steroid metabolic process
salmon_5H	positive regulation of vasoconstriction<br>ethanolamine-containing compound metabolic process<br>protein targeting to mitochondrion
tan_24	Golgi stack<br>chromosome organization<br>macromolecule methylation<br>mitotic sister chromatid segregation<br>methylation<br>chromatin modification
tan_5H	cellular response to heat<br>neuromuscular synaptic transmission<br>lipoprotein biosynthetic process<br>lipoprotein metabolic process<br>MAPK import into nucleus<br>RNA import into nucleus
turquoise_20	oxidoreductase activity<br>negative regulation of defense response<br>flavin adenine dinucleotide binding<br>regulation of natural killer cell mediated immunity<br>positive regulation of natural killer cell mediated immunity<br>regulation of histone deacetylation
turquoise_24	leading edge membrane<br>negative regulation of protein catabolic process<br>response to external stimulus<br>positive chemotaxis<br>cellular component movement<br>neuron projection membrane
turquoise_5H	DNA catabolic process<br>axoneme<br>SCF ubiquitin ligase complex<br>TBP-class protein binding<br>regulated secretory pathway<br>transcription initiation from RNApolII promoter
turquoise_U	polysaccharide catabolic process<br>glycogen catabolic process<br>glucan catabolic process<br>cellular polysaccharide catabolic process<br>polysaccharide metabolic process<br>methylated histone residue binding
yellow_20	transcription from RNApolII promoter<br>hormone biosynthetic process<br>vascular endothelial growth factor production<br>regulation of vascular endothelial growth factor<br>regulation of transcription from RNApolII promoter

Continued on next page

Table 1 – *Continued from previous page*

Module	GO Term
	positive regulation of protein import into nucleus (translocation)
yellow_24	proteasomal protein catabolic process<br>proteasomal ubiquitin-dependent protein catabolic process<br>cell-cell adhesion involved in gastrulation<br>negative regulation of gene expression<br>transcription cofactor activity<br>negative regulation of macromolecule biosynthetic process
yellow_5H	chromatin DNA binding<br>thyroid hormone receptor activator activity<br>positive regulation of response to interferon-gamma<br>positive regulation of interferon-gamma-mediated signaling pathway<br>electron carrier activity<br>androgen metabolic process
yellow_U	BRCA1-A complex<br>photoreceptor cell maintenance<br>positive regulation of response to DNA damage stimulus<br>visual perception<br>ion channel binding<br>calcium-dependent phospholipid binding

REFERENCES

Allen, P. B., Hvalby, Ø., Jensen, V., Errington, M. L., Ramsay, M., Chaudhry, F. A., et al. (2000), Protein phosphatase-1 regulation in the induction of long-term potentiation: heterogeneous molecular mechanisms, *The Journal of Neuroscience*, 20, 10, 3537–3543

Brakeman, P., Lanahan, A., O'Brien, R., Roche, K., Barnes, C., Huganir, R., et al. (1997), Homer: a protein that selectively binds metabotropic glutamate receptors, *Nature*, 386, 284–288

Bruel-Jungerman, E., Davis, S., Rampon, C., and Laroche, S. (2006), Long-term potentiation enhances neurogenesis in the adult dentate gyrus, *The Journal of neuroscience*, 26, 22, 5888–5893

Chen, W. Y., Wang, D. H., Yen, R. C., Luo, J., Gu, W., and Baylin, S. B. (2005), Tumor suppressor hic1 directly regulates sirt1 to modulate p53-dependent dna-damage responses, *Cell*, 123, 3, 437–448

Cole, A. J., Saffen, D. W., Baraban, J. M., and Worley, P. F. (1989), Rapid increase of an immediate early gene messenger rna in hippocampal neurons by synaptic nmda receptor activation, *Nature*, 340, 6233, 474–476

Contractor, A., Rogers, C., Maron, C., Henkemeyer, M., Swanson, G. T., and Heinemann, S. F. (2002), Trans-synaptic eph receptor-ephrin signaling in hippocampal mossy fiber ltp, *Science*, 296, 5574, 1864–1869

Dayer, A. G., Ford, A. A., Cleaver, K. M., Yassaee, M., and Cameron, H. A. (2003), Short-term and long-term survival of new neurons in the rat dentate gyrus, *Journal of Comparative Neurology*, 460, 4, 563–572

Engert, F. and Bonhoeffer, T. (1999), Dendritic spine changes associated with hippocampal long-term synaptic plasticity, *Nature*, 399, 6731, 66–70

Fischer, M., Kaech, S., Knutti, D., and Matus, A. (1998), Rapid actin-based plasticity in dendritic spines, *Neuron*, 20, 5, 847–854

Freudenthal, R. and Romano, A. (2000), Participation of rel/nf-κb transcription factors in long-term memory in the crab chasmagnathus, *Brain research*, 855, 2, 274–281

Freudenthal, R., Romano, A., and Routtenberg, A. (2004), Transcription factor nf-kb activation after in vivo perforant path ltp in mouse hippocampus, *Hippocampus*, 14, 6, 677–683

Fukazawa, Y., Saitoh, Y., Ozawa, F., Ohta, Y., Mizuno, K., and Inokuchi, K. (2003), Hippocampal ltp is accompanied by enhanced f-actin content within the dendritic spine that is essential for late ltp maintenance in vivo, *Neuron*, 38, 3, 447–460

Gould, E., Beylin, A., Tanapat, P., Reeves, A., and Shors, T. J. (1999), Learning enhances adult neurogenesis in the hippocampal formation, *Nature neuroscience*, 2, 3, 260–265

Greengard, P., Allen, P. B., and Nairn, A. C. (1999), Beyond the dopamine receptor: the darpp-32/protein phosphatase-1 cascade, *Neuron*, 23, 3, 435–447

Guan, J.-S., Haggarty, S. J., Giacometti, E., Dannenberg, J.-H., Joseph, N., Gao, J., et al. (2009), Hdac2 negatively regulates memory formation and synaptic plasticity, *Nature*, 459, 7243, 55–60

Haber, D. A., Sohn, R. L., Buckler, A. J., Pelletier, J., Call, K. M., and Housman, D. E. (1991), Alternative splicing and genomic structure of the wilms tumor gene wt1, *Proceedings of the National Academy of Sciences*, 88, 21, 9618–9622

Hanai, Y., Tokuda, H., Ohta, T., Matsushima-Nishiwaki, R., Takai, S., and Kozawa, O. (2006), Phosphatidylinositol 3-kinase/akt auto-regulates pdgf-bb-stimulated interleukin-6 synthesis in osteoblasts, *Journal of cellular biochemistry*, 99, 6, 1564–1571

Håvik, B., Røkke, H., Dagyte, G., Stavrum, A., Bramham, C., and Steen, V. (2007), Synaptic activity-induced global gene expression patterns in the dentate gyrus of adult behaving rats: Induction of immunity-linked genes, *Neuroscience*, 148, 4, 925–936

Joilin, G., Guévremont, D., Ryan, B., Claudianos, C., Cristino, A. S., Abraham, W. C., et al. (2014), Rapid regulation of microrna following induction of long-term potentiation in vivo, *Frontiers in molecular neuroscience*, 7

Karpova, A., Sanna, P., and Behnisch, T. (2006), Involvement of multiple phosphatidylinositol 3-kinase-dependent pathways in the persistence of late-phase long term potentiation expression, *Neuroscience*, 137, 3, 833–841

Kashishian, A., Kazlauskas, A., and Cooper, J. A. (1992), Phosphorylation sites in the pdgf receptor with different specificities for binding gap and pi3 kinase in vivo, *The EMBO journal*, 11, 4, 1373

Kato, A., Ozawa, F., Saitoh, Y., Hirai, K., and Inokuchi, K. (1997), vesl, a gene encoding vasp/ena family related protein, is upregulated during seizure, long-term potentiation and synaptogenesis, *FEBS letters*, 412, 1, 183–189

Kato, A. S., Gill, M. B., Ho, M. T., Yu, H., Tu, Y., Siuda, E. R., et al. (2010), Hippocampal ampa receptor gating controlled by both tarp and cornichon proteins, *Neuron*, 68, 6, 1082–1096

Kim, C.-H. and Lisman, J. E. (1999), A role of actin filament in synaptic transmission and long-term potentiation, *The Journal of neuroscience*, 19, 11, 4314–4324

Klein, R. (2008), Bidirectional modulation of synaptic functions by eph/ephrin signaling, *Nature neuroscience*, 12, 1, 15–20

Krucker, T., Siggins, G. R., and Halpain, S. (2000), Dynamic actin filaments are required for stable long-term potentiation (ltp) in area ca1 of the hippocampus, *Proceedings of the National Academy of Sciences*, 97, 12, 6856–6861

Kruijer, W., Cooper, J. A., Hunter, T., and Verma, I. M. (1983), Platelet-derived growth factor induces rapid but transient expression of the c-fos gene and protein, *Nature*, 312, 5996, 711–716

Levenson, J. M., Roth, T. L., Lubin, F. D., Miller, C. A., Huang, I.-C., Desai, P., et al. (2006), Evidence that dna (cytosine-5) methyltransferase regulates synaptic plasticity in the hippocampus, *Journal of Biological Chemistry*, 281, 23, 15763–15773

Lisman, J. E. (1985), A mechanism for memory storage insensitive to molecular turnover: a bistable autophosphorylating kinase, *Proceedings of the National Academy of Sciences*, 82, 9, 3055–3057

Liu, T., Liu, P. Y., and Marshall, G. M. (2009), The critical role of the class iii histone deacetylase sirt1 in cancer, *Cancer research*, 69, 5, 1702–1705

Lubin, F. D., Roth, T. L., and Sweatt, J. D. (2008), Epigenetic regulation of bdnf gene transcription in the consolidation of fear memory, *The Journal of Neuroscience*, 28, 42, 10576–10586

Maletic-Savatic, M., Malinow, R., and Svoboda, K. (1999), Rapid dendritic morphogenesis in ca1 hippocampal dendrites induced by synaptic activity, *Science*, 283, 5409, 1923–1927

Man, H.-Y., Wang, Q., Lu, W.-Y., Ju, W., Ahmadian, G., Liu, L., et al. (2003), Activation of pi3-kinase is required for ampa receptor insertion during ltp of mepscs in cultured hippocampal neurons, *Neuron*, 38, 4, 611–624

Matsuzaki, M., Honkura, N., Ellis-Davies, G. C., and Kasai, H. (2004), Structural basis of long-term potentiation in single dendritic spines, *Nature*, 429, 6993, 761–766

Matus, A. (2000), Actin-based plasticity in dendritic spines, *Science*, 290, 5492, 754–758

Meberg, P. J., Kinney, W. R., Valcourt, E. G., and Routtenberg, A. (1996), Gene expression of the transcription factor nf-kb in hippocampus: regulation by synaptic activity, *Molecular brain research*, 38, 2, 179–190

Merlo, E., Freudenthal, R., and Romano, A. (2002), The iκb kinase inhibitor sulfasalazine impairs long-term memory in the crab chasmagnathus, *Neuroscience*, 112, 1, 161–172

Michán, S., Li, Y., Chou, M. M.-H., Parrella, E., Ge, H., Long, J. M., et al. (2010), Sirt1 is essential for normal cognitive function and synaptic plasticity, *The Journal of Neuroscience*, 30, 29, 9695–9707

Miller, C. A. and Sweatt, J. D. (2007), Covalent modification of dna regulates memory formation, *Neuron*, 53, 6, 857–869

Muller, D., Toni, N., and Buchs, P.-A. (2000), Spine changes associated with long-term potentiation, *Hippocampus*, 10, 5, 596–604

Nishizuka, Y. (1995), Protein kinase c and lipid signaling for sustained cellular responses., *The FASEB Journal*, 9, 7, 484–496

Niu, S., Yabut, O., and D'Arcangelo, G. (2008), The reelin signaling pathway promotes dendritic spine development in hippocampal neurons, *The Journal of Neuroscience*, 28, 41, 10339–10348

Opazo, P., Watabe, A. M., Grant, S. G., and O'Dell, T. J. (2003), Phosphatidylinositol 3-kinase regulates the induction of long-term potentiation through extracellular signal-related kinase-independent mechanisms, *The Journal of neuroscience*, 23, 9, 3679–3688

Owens, D. and Keyse, S. (2007), Differential regulation of map kinase signalling by dual-specificity protein phosphatases, *Oncogene*, 26, 22, 3203–3213

Qiu, S., Zhao, L. F., Korwek, K. M., and Weeber, E. J. (2006), Differential reelin-induced enhancement of nmda and ampa receptor activity in the adult hippocampus, *The Journal of neuroscience*, 26, 50, 12943–12955

Raber, J., Rola, R., LeFevour, A., Morhardt, D., Curley, J., Mizumatsu, S., et al. (2004), Radiation-induced cognitive impairments are associated with changes in indicators of hippocampal neurogenesis, *Radiation research*, 162, 1, 39–47

Rice, D. S. and Curran, T. (2001), Role of the reelin signaling pathway in central nervous system development, *Annual review of neuroscience*, 24, 1, 1005–1039

Richardson, C., Tate, W., Mason, S., Lawlor, P., Dragunow, M., and Abraham, W. (1992), Correlation between the induction of an immediate early gene, zif/268, and long-term potentiation in the dentate gyrus, *Brain research*, 580, 1, 147–154

Ryan, M. M., Mason-Parker, S. E., Tate, W. P., Abraham, W. C., and Williams, J. M. (2011), Rapidly induced gene networks following induction of long-term potentiation at perforant path synapses in vivo, *Hippocampus*, 21, 5, 541–553

Sala, C., Piëch, V., Wilson, N. R., Passafaro, M., Liu, G., and Sheng, M. (2001), Regulation of dendritic spine morphology and synaptic function by shank and homer, *Neuron*, 31, 1, 115–130

Sanna, P. P., Cammalleri, M., Berton, F., Simpson, C., Lutjens, R., Bloom, F. E., et al. (2002), Phosphatidylinositol 3-kinase is required for the expression but not for the induction or the maintenance of long-term potentiation in the hippocampal ca1 region, *The Journal of neuroscience*, 22, 9, 3359–3365

Sarkisov, D. V. and Wang, S. S.-H. (2008), Order-dependent coincidence detection in cerebellar purkinje neurons at the inositol trisphosphate receptor, *The Journal of Neuroscience*, 28, 1, 133–142

Shiraishi-Yamaguchi, Y. and Furuichi, T. (2007), The homer family proteins, *Genome Biol*, 8, 2, 206

Shors, T. J., Miesegaes, G., Beylin, A., Zhao, M., Rydel, T., and Gould, E. (2001), Neurogenesis in the adult is involved in the formation of trace memories, *Nature*, 410, 6826, 372–376

Snyder, J., Hong, N., McDonald, R., and Wojtowicz, J. (2005), A role for adult neurogenesis in spatial long-term memory, *Neuroscience*, 130, 4, 843–852

Tiberi, L., Van Den Ameele, J., Dimidschstein, J., Piccirilli, J., Gall, D., Herpoel, A., et al. (2012), Bcl6 controls neurogenesis through sirt1-dependent epigenetic repression of selective notch targets, *Nature neuroscience*, 15, 12, 1627–1635

Weeber, E. J., Beffert, U., Jones, C., Christian, J. M., Förster, E., Sweatt, J. D., et al. (2002), Reelin and apoe receptors cooperate to enhance hippocampal synaptic plasticity and learning, *Journal of Biological Chemistry*, 277, 42, 39944–39952

Wigström, H. and Gustafsson, B. (1983), Facilitated induction of hippocampal long-lasting potentiation during blockade of inhibition, *Nature*, 301, 603–604

Yokote, K., Mori, S., Hansen, K., McGlade, J., Pawson, T., Heldin, C.-H., et al. (1994), Direct interaction between shc and the platelet-derived growth factor beta-receptor, *Journal of Biological Chemistry*, 269, 21, 15337–15343

Ziv, N. E. and Smith, S. J. (1996), Evidence for a role of dendritic filopodia in synaptogenesis and spine formation, *Neuron*, 17, 1, 91–102
